# Community-acquired pneumonia on the intensive care unit: secondary analysis of 17,869 cases in the ICNARC Case Mix Programme Database

**DOI:** 10.1186/cc4927

**Published:** 2006-06-16

**Authors:** Mark Woodhead, Catherine A Welch, David A Harrison, Geoff Bellingan, Jon G Ayres

**Affiliations:** 1 Manchester Royal Infirmary, Oxford Road, Manchester M13 9WL, UK; 2 Intensive Care National Audit & Research Centre (ICNARC), Tavistock House, Tavistock Square, London WC1H 9HR, UK; 3 Critical Care Directorate, University College Hospital, Euston Road, London NW1 2BU, UK; 4 Department of Environmental and Occupational Medicine, Liberty Safe Work Research Centre, University of Aberdeen, Foresterhill Road, Aberdeen AB25 2ZP, UK

## Abstract

**Introduction:**

This paper describes the case mix, outcome and activity for admissions to intensive care units (ICUs) with community-acquired pneumonia (CAP).

**Methods:**

We conducted a secondary analysis of a high quality clinical database, the Intensive Care National Audit & Research Centre (ICNARC) Case Mix Programme Database, of 301,871 admissions to 172 adult ICUs across England, Wales and Northern Ireland, 1995 to 2004. Cases of CAP were identified from pneumonia admissions excluding nosocomial pneumonias and the immuno-compromised. It was not possible to review data from the time of hospital admission; therefore, some patients who developed hospital-acquired/nosocomial pneumonia may have been included.

**Results:**

We identified 17,869 cases of CAP (5.9% of all ICU admissions). There was a 128% increase in admissions for CAP from 12.8 per unit to 29.2 per unit during the study period compared to only a 24% rise in total ICU admissions (*p* < 0.001). Eighty-five percent of admissions were from within the same hospital. Fifty-nine percent of cases were admitted to the ICU <2 days, 21.5% between 2 and 7 days, and 19.5% >7 days after hospital admission. Between 1995 and 1999 and 2000 and 2004 there was a rise in admissions from accident and emergency (14.8% to 16.8%; *p* < 0.001) and high dependency units (6.9% to 11.9%; *p* < 0.001) within the same hospital, those aged >74 (18.5 to 26.1%; *p* < 0.001), and mean APACHE II score (6.83 to 6.91; *p* < 0.001). There was a fall in past history of severe respiratory problems (8.7% to 6.4%; *p* < 0.001), renal replacement therapy (1.6% to 1.2%; *p* < 0.01), steroid treatment (3.4% to 2.8%; *p* < 0.05), sedation/paralysis (50.2% to 40.4%; *p* < 0.001), cardiopulmonary resuscitation prior to admission (7.5% to 5.5%; *p* < 0.001), and septic shock (7.3% to 6.6%; *p* < 0.001). ICU mortality was 34.9% and ultimate hospital mortality 49.4%. Mortality was 46.3% in those admitted to the ICU within 2 days of hospital admission rising to 50.4% in those admitted at 2 to 7 days and 57.6% in those admitted after 7 days following hospital admission.

**Conclusion:**

CAP makes up a small, but important and rising, proportion of adult ICU admissions. Survival of over half of all cases vindicates the use of ICU facilities in CAP management. Nevertheless, overall mortality remains high, especially in those admitted later in their hospital stay.

## Introduction

Community-acquired pneumonia (CAP) is an increasingly common reason for hospital admission [1]. The majority of such admissions recover uneventfully with appropriate management. Between 5% and 10% [2] are more severely ill and are managed on an intensive care unit (ICU), up to 58% [3] of whom go on to die. There have been four UK studies of such patients [3-6]. While producing useful information, these studies are limited by their small size (each <100 cases), location in a single institution [3-5] and being out of date in relation to recent changes in clinical practice. Changes in population demographics, acute hospital admission practices, ICU admission practices (linked to the absence of an agreed definition of what constitutes severe CAP), and introduction of new interventions (for example, non-invasive ventilation) suggest that a further assessment of CAP on the ICU is worthwhile.

We have taken the opportunity provided by the Intensive Care National Audit & Research Centre (ICNARC) Case Mix Programme (CMP) Database to study nearly 18,000 such cases managed on 172 ICUs between 1995 and 2004 with details of case mix and outcome presented here.

## Materials and methods

### Case Mix Programme Database

The CMP is a national comparative audit of adult, general critical care units in England, Wales and Northern Ireland co-ordinated by ICNARC. Data were extracted for 301,871 admissions to 172 ICUs from the CMP Database, covering the period December 1995 to June 2004. Details of the data collection and validation have been reported previously [7].

The CMP has approval from the Patient Information Advisory Group to hold identifiable data without consent. Ethics committee approval was not required.

### Selection of cases

Information on the reason for admission to the CMP unit is recorded in the CMP Database using a standard coding method, the ICNARC Coding Method [8].

**Figure 1:**
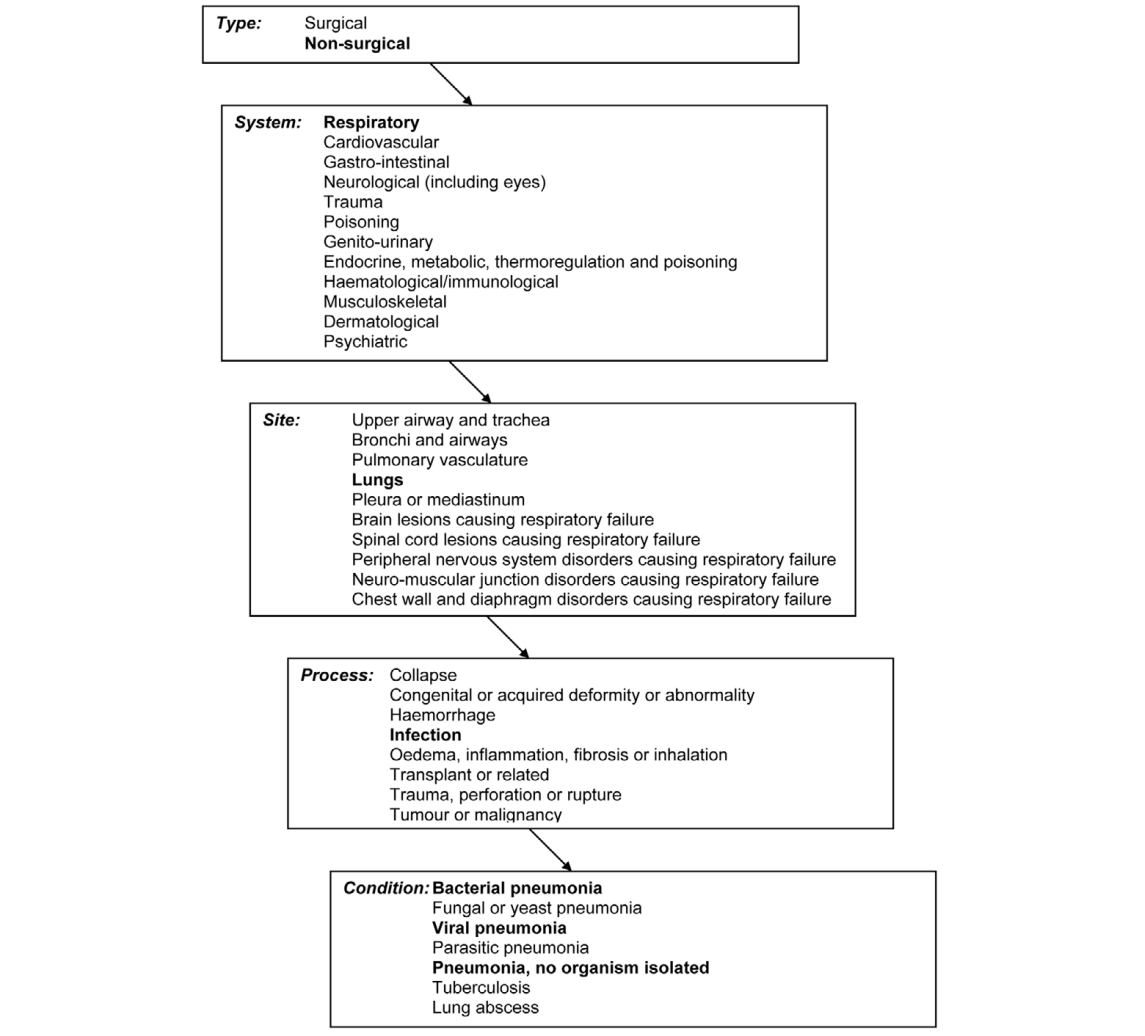
The Intensive Care National Audit & Research Centre Coding Method for pneumonia.

Admissions were selected as having probable pneumonia if the recording of the primary reason for admission, or ultimate primary reason for admission if the diagnosis changed after 24 hours, was any of 'Bacterial pneumonia', 'Viral pneumonia' or 'Pneumonia, no organism isolated' (Figure 1). Admissions whose ultimate primary reason for admission to the CMP unit, based on information known after the first 24 hours in the CMP unit, was not one of the above (that is to say, the primary reason for admission was corrected) were excluded.

Community-acquired pneumonia is not coded separately, so cases were identified by the exclusion of pneumonia cases that were likely to be nosocomial or occurring in a previously immuno-compromised patient. Likely nosocomial pneumonia was defined as surgical admission or transfer from another ICU more than two days after the original hospital admission. Surgical admissions were identified as those with a reason for admission to the CMP unit that was surgical, or a source of admission or location immediately prior to source of admission of theatre and recovery in the hospital. Immuno-compromised patients were identified by the recording of any of the following in the past medical history: AIDS, radiotherapy, chemotherapy, metastatic disease, acute myelogenous leukaemia or acute lymphocytic leukaemia or multiple myeloma, chronic myelogenous leukaemia or chronic lymphocytic leukaemia, lymphoma, or congenital immunohumoral or cellular immune deficiency state.

### Data

Data were extracted on the case mix, outcome and activity as defined below. Microbiological and antibiotic management data are not included in the dataset and thus could not be analysed.

#### Case mix

Age, gender, primary and secondary reasons for admission, cardiopulmonary resuscitation (CPR) within 24 hours prior to admission, and systolic blood pressure, heart rate, and mechanical ventilation at admission were extracted.

The following physiological variables were extracted from the first 24 hours in the CMP unit: sedated or paralysed and sedated for whole of first 24 hours, lowest total Glasgow Coma score if not sedated/paralysed, lowest arterial oxygen tension (PaO_2_), lowest pH and associated arterial carbon dioxide tension (PaCO_2_).

Acute severity was measured with the Acute Physiology and Chronic Health Evaluation (APACHE) II Acute Physiology Score (APS) and the APACHE II score [9]; the former encompasses a weighting for acute physiology (defined by derangement from the normal range for 12 physiological variables in the first 24 hours in the ICU); the latter additionally encompasses a weighting for age and for a past medical history of specified serious conditions.

#### Outcome

Survival data were extracted at discharge from the CMP unit and at ultimate discharge from hospital.

#### Activity

Length of hospital stay prior to admission to the CMP unit was calculated from the dates of admission to the hospital and admission to the unit. Length of stay in the CMP unit was calculated in fraction of days from the dates and times of admission and discharge or death. Total length of stay in hospital was calculated in days from the dates of original admission and ultimate discharge or death.

Readmissions to the CMP unit within the same hospital stay were identified from the postcode, date of birth and sex, and confirmed by the participating units.

### Analyses

Case mix, outcome and activity were described for all admissions identified as probable CAP and separately for admissions during the periods 1995 to 1999 and 2000 to 2004.

The demographics and first 24 hour physiology were compared by length of stay in hospital prior to ICU admission in three groups: those admitted to ICU within two days of hospital admission, those admitted to ICU between two and seven days following hospital admission, and those admitted to ICU more than seven days after hospital admission.

## Results

### Case mix

The definition of CAP was fulfilled by 17,869 cases representing 5.9% of all ICU admissions during the study period. Of these, 10,271 (57.5%) cases were male with 2,941 (16.5%) aged less than 45 years and 4,257 (23.9%) aged 75 years or older (Table 1). Fifty-nine percent of cases were admitted to the ICU less than two days, 21.5% between two and seven days and 19.5% more than seven days after hospital admission. Eighty-five per cent of admissions were from within the same hospital, but a significant minority of 15% were transferred from another hospital. There were 1,080 cases (6.1%) only admitted after receiving cardiopulmonary resuscitation and 9,751 (54.6%) were mechanically ventilated at admission with 7,737 (43.3%) sedated or paralysed and sedated for the whole of the first 24 hours.

**Table 1:** Case mix for admissions with community-acquired pneumonia, overall and by year of admission: 1995 to 1999 versus 2000 to 2004

	All admissions	Admissions 1995 to 1999	Admissions 2000 to 2004	P value
Admissions, n (%)	17,869 (5.55)	5,333 (1.65)	12,536 (3.89)	
Mean (SD) age, years	61.6 (17.6)	60.2 (17.5)	62.2 (17.6)	<0.001
Age, n (%)				
<16 years	336 (1.9)	116 (2.2)	220 (1.8)	
16 to 24 years	475 (2.7)	144 (2.7)	331 (2.6)	
25 to 34 years	830 (4.6)	298 (5.6)	532 (4.2)	
35 to 44 years	1,300 (7.3)	385 (7.2)	915 (7.3)	
45 to 54 years	2,184 (12.2)	686 (12.8)	1,498 (12.0)	
55 to 64 years	3,453 (19.3)	1,087 (20.4)	2,366 (18.9)	
65 to 74 years	5,034 (28.2)	1,632 (30.6)	3,402 (27.1)	
75 to 84 years	3,837 (21.5)	916 (17.2)	2,921 (23.3)	
85+ years	420 (2.4)	69 (1.3)	351 (2.8)	
Gender male, n (%)	10,271 (57.5)	3,067 (57.5)	7,204 (57.5)	0.954
Source of admission, n (%)				<0.001
Ward, same hospital	9,583 (53.6)	2,941 (55.2)	6,645 (53.0)	
A&E, same hospital, clinic or home	2,888 (16.2)	788 (14.8)	2,100 (16.8)	
Other intermediate care area, same hospital	649 (3.6)	162 (3.0)	487 (3.9)	
HDU, same hospital	1,867 (10.5)	370 (6.9)	1,497 (11.9)	
ICU, same hospital	198 (1.1)	77 (1.5)	121 (1.0)	
ICU, other hospital	1,355 (7.6)	474 (8.9)	881 (7.0)	
HDU, other hospital	157 (0.9)	44 (0.8)	113 (0.9)	
Other hospital (not ICU or HDU)	1,169 (6.5)	477 (8.9)	692 (5.5)	
Past medical history, n (%)				
Severe liver problems	219 (1.2)	58 (1.1)	161 (1.3)	0.274
Very severe cardiovascular disease	334 (1.9)	96 (1.8)	238 (1.9)	0.656
Steroid treatment	535 (3.0)	183 (3.4)	352 (2.8)	0.025
Severe respiratory problems	1,265 (7.1)	465 (8.7)	800 (6.4)	<0.001
Chronic renal replacement therapy	231 (1.3)	87 (1.6)	144 (1.2)	0.009
Primary reason for admission, n (%)				<0.001
Viral pneumonia	361 (2.0)	164 (3.1)	197 (1.6)	
Bacterial pneumonia	6,972 (49.0)	2,270 (42.6)	4,702 (37.5)	
Pneumonia, no organism isolated	10,536 (59.0)	2,899 (54.3)	7,637 (60.9)	
Secondary reason for admission, n (%)				<0.001
Pre-existing				
Obstructive airways disease	1,309 (7.3)	423 (7.9)	886 (7.1)	
Trauma	119 (0.7)	32 (0.6)	87 (0.7)	
Diabetes mellitus	117 (0.7)	49 (0.9)	68 (0.5)	
Chronic neuromuscular disorders	99 (0.6)	32 (0.6)	67 (0.5)	
Alcoholic cirrhosis	124 (0.7)	24 (0.5)	100 (0.8)	
Chronic renal failure	184 (1.0)	49 (0.9)	135 (1.1)	
Pulmonary fibrosis or fibrosing alveolitis	112 (0.6)	25 (0.5)	87 (0.7)	
Asthma attack	176 (1.0)	62 (1.2)	114 (0.9)	
Inhalation pneumonitis (gastrointestinal contents)	56 (0.3)	12 (0.2)	44 (0.4)	
Occurring as CAP complication				
Septic shock/septicaemia	1,223 (6.8)	389 (7.3)	834 (6.6)	
Pleural effusion	122 (0.7)	17 (0.3)	105 (0.8)	
Could be pre-existing or complication				
Acute renal failure	877 (4.9)	238 (4.5)	639 (5.1)	
Cardiac dysrhythmia	246 (1.4)	58 (1.1)	188 (1.5)	
Cardiac failure	643 (3.6)	213 (4.0)	430 (3.4)	
Cardiogenic shock	59 (0.3)	22 (0.4)	37 (0.3)	
Acute myocardial infarction	218 (1.2)	55 (1.0)	163 (1.3)	
Pulmonary embolus (thrombus)	55 (0.3)	23 (0.4)	32 (0.3)	
Status epilepticus or uncontrolled seizures	91 (0.5)	32 (0.6)	59 (0.5)	
Non-cardiogenic pulmonary oedema (ARDS)	152 (0.9)	45 (0.8)	107 (0.9)	
Other/none	11,887 (66.5)	3,533 (66.3)	8,354 (66.6)	
CPR prior to admission, n (%)	1,080 (6.1)	396 (7.5)	684 (5.5)	<0.001
Mean (SD) SBP at admission, mmHg	123.8 (30.9)	123.5 (32.2)	123.9 (30.4)	0.486
Mean (SD) heart rate at admission, bpm	108.6 (24.6)	110.1 (24.8)	108.0 (24.5)	<0.001
Mechanically ventilated at admission, n (%)	9,751 (54.6)	3,009 (56.4)	6,742 (53.8)	0.001
Sedated or paralysed and sedated for whole of first 24 hours, n (%)	7,737 (43.3)	2,676 (50.2)	5,061 (40.4)	<0.001
Median (IQR) lowest total GCS if not sedated/paralysed	15 (11–15)	15 (11–15)	15 (11–15)	0.249
Median (IQR) highest non-ventilated respiratory rate, breaths/minute	32 (26–40)	32 (25–40)	33 (26–40)	0.030
Median (IQR) lowest PaO_2_, kPa	9.0 (7.7–10.4)	9.1 (7.7–10.6)	8.9 (7.7–10.4)	0.023
Median (IQR) lowest pH	7.3 (7.2–7.4)	7.3 (7.2–7.4)	7.3 (7.2–7.4)	0.002
Median (IQR) associated PaCO_2_, kPa	6.7 (5.3–8.6)	6.5 (5.2–8.3)	6.7 (5.2–8.3)	<0.001
Mean (SD) APACHE II APS^a^	15.31 (6.34)	15.09 (6.25)	15.40 (6.38)	0.003
Mean (SD) APACHE II score^a^	19.63 (6.89)	19.37 (6.83)	19.74 (6.91)	<0.001

^a^APACHE II exclusions: age <16 years; ICU stay <8 hours; readmissions within same hospital stay; transfers from another ICU; admissions following coronary artery bypass grafting; admissions for primary burns. A&E, accident and emergency; APACHE, Acute Physiology and Chronic Health Evaluation; APS, Acute Physiology Score; ARDS, acute respiratory distress syndrome; CAP, community-acquired pneumonia; CPR, cardiopulmonary resuscitation; GCS, Glasgow Coma score; HDU, high dependency unit; ICU, intensive care unit; IQR, interquartile range; PaCO_2_, arterial carbon dioxide tension; PaO_2_, arterial oxygen tension; SBP, systolic blood pressure; SD, standard deviation.

Past medical problems were infrequently recorded, with severe respiratory problems featuring most frequently, but in only 7.1%. It is likely that these figures are low due to the restricted definition of severe respiratory problems ('permanent shortness of breath with light activity, e.g., walking 20 m'), but under-recording cannot be excluded. Similarly, secondary reasons for ICU admission suggested low rates of chronic diseases, which may also be an underestimate of their true frequency. Of other secondary reasons for ICU admission, septic shock (6.8%) and pleural effusion (0.7%) are likely to have occurred as a complication of CAP, but others such as acute renal failure and myocardial infarction may have been a complication of or complicated by the CAP. All were infrequently recorded (<5%).

### Outcome and activity

ICU mortality was 34.9% and ultimate hospital mortality 49.4% (Table 2). Of 8,609 total deaths, 2,381 (27.7%) occurred after leaving the ICU. Median (interquartile range) length of stay in the ICU was: survivors 6 (3 to 14) days; non-survivors 4 (1 to 10) days (Table 3). Median (interquartile range) length of stay in hospital was: survivors 30 (17 to 53) days; non-survivors 12 (4 to 26) days (Table 3).

**Table 2:** Outcome for admissions with community-acquired pneumonia, overall and by year of admission: 1995 to 1999 versus 2000 to 2004

	All admissions		Admissions 1995 to 1999		Admissions 2000 to 2004		P value
	N	%	n	%	n	%	

Mortality in CMP unit	6,228	(34.9)	1,757	(33.0)	4,471	(35.7)	<0.001
Ultimate hospital mortality^a^	8,609	(49.4)	2,484	(47.7)	6,125	(50.1)	0.004

^a^Excluding readmissions within the same hospital stay. CMP, Case Mix Programme.

**Table 3:** Activity for admissions with community-acquired pneumonia, overall and by year of admission: 1995 to 1999 versus 2000 to 2004

	All admissions	Admissions 1995 to 1999	Admissions 2000 to 2004	P value
Length of stay prior to admission to the CMP unit, days	2 (0–7)	1 (0–6)	2 (0–7)	<0.001
Length of stay in CMP unit, days				
Survivors	6 (3–14)	6 (3–13)	6 (3–14)	0.871
Non-survivors	4 (1–10)	4 (1–11)	4 (1–9)	0.098
Total length of stay in hospital^a^, days				
Survivors	30 (17–53)	28 (16–51)	31 (17–55)	<0.001
Non-survivors	12 (4–26)	12 (4–27)	12 (4–26)	0.749

Values are median (interquartile range). ^a^Excluding readmissions within the same hospital stay. CMP, Case Mix Programme.

### Comparison of those admitted to ICU <2 days, 2 to 7 days and >7 days after hospital admission

As time from hospital admission lengthened, the case mix altered (Additional data file 1) with a rise in the proportion of older (>55 years) and male cases admitted. The proportion admitted from a ward, including high dependency units (HDUs), rose while those admitted from accident and emergency and those admitted after CPR fell. There were rises in the proportions with a past history of severe liver problems and chronic renal replacement therapy, but a fall in those with severe respiratory problems. Markers of respiratory failure in the non-ventilated became more frequent (higher respiratory rates and PaCO_2_) and abnormal serum sodium was more common. However, there was a reduction in the frequency of other abnormal biochemical variables, with abnormal pH, base excess, potassium, urea and creatinine being less common with time. Mortality was 46.3% in those admitted to the ICU <2 days after admission rising to 50.4% in those admitted at 2 to 7 days and 57.6 in those only admitted after 7 days in hospital (*p* < 0.001).

### Comparison of 1995 to 1999 with 2000 to 2004

**Figure 2:**
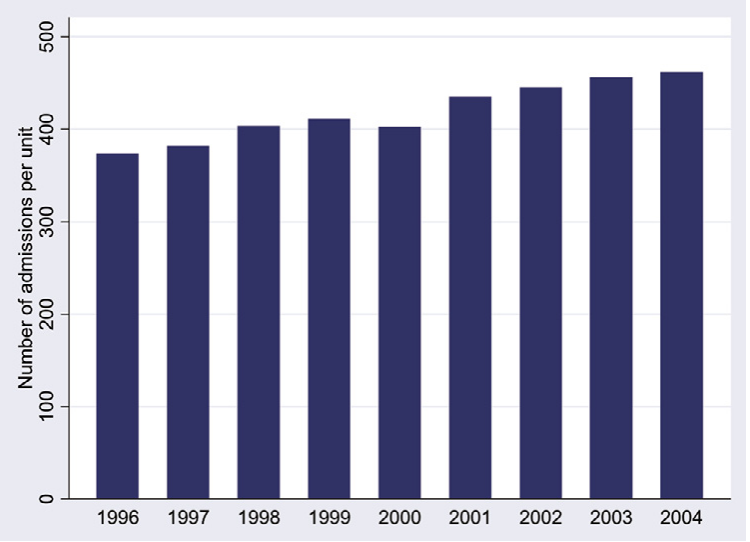
Number of admissions to an intensive care unit per unit each year.

**Figure 3:**
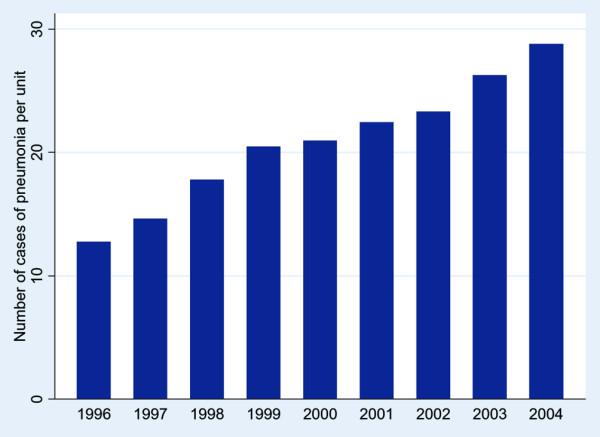
Number of admissions to an intensive care unit with community acquired pneumonia per unit each year.

The total number of admissions per unit rose by 24% throughout the study period (Figure 2); however, the number of CAP ICU admissions rose by 128% (Figure 3) from 12.8 per unit in 1996 to 29.2 per unit in 2004 (*p* < 0.001). The proportion of admissions from other hospitals did not change, but admission from within the same hospital from HDU (6.9 to 11.9%) and accident and emergency (14.8 to 16.8%) rose (*p* < 0.001). Between the two periods there was a rise in those aged >74 years (18.5 to 26.1%; *p* < 0.001) and mean APACHE II score (6.83 to 6.91; *p* < 0.001), and a fall in past history of severe respiratory problems (8.7 to 6.4%; *p* < 0.001), renal replacement therapy (1.6 to 1.2%; *p* < 0.01), steroid treatment (3.4 to 2.8%; *p* < 0.05), those sedated and paralysed at admission (50.2 to 40.4%; *p* < 0.001), CPR prior to admission (7.5 to 5.5%; *p* < 0.001) and septic shock (7.3 to 6.6%; *p* < 0.001). Death rates rose slightly between the two periods (ICU mortality 33 to 35.7%, *p* < 0.001; hospital mortality 47.7 to 50.1%, *p* < 0.005). Median (interquartile range) hospital stay in survivors rose between the two periods (28 (16 to 51) to 31 (17 to 55); *p* < 0.001).

## Discussion

The main findings of this study, the largest study of CAP admissions to ICU published worldwide, are of the progressive rise in the number of admissions with time and the survival of half of all cases. While CAP accounts for only a small proportion of total ICU admissions, the rise in the number of CAP cases has been disproportionately large in comparison to the rise in ICU admissions overall. Illness severity, whether judged by admission gas exchange parameters, APACHE score, septic shock, length of hospital stay or mortality, does not appear to have altered sufficiently to explain this large increase, suggesting that the rise is either due to an increase in the total number of severely ill CAP cases presenting to hospital or the accommodation of a previously unmet need. There is evidence to support both explanations and it is likely that a combination of the two has contributed. In support of the former is the disproportionate rise in those aged >74 years (which could also be due to previous unmet need) and the increase in overall hospital admissions for CAP [1]. The previously unmet need theory is supported by the fall in the proportion that are paralysed and sedated on ICU arrival, perhaps reflecting a recent increase in the use of non-invasive ventilation (although this was not specifically recorded in the database). Statistically significant, but clinically small, rises/changes in admission source, steroid treatment, renal replacement therapy, severe respiratory problems, pneumonia type, and secondary admission reason are unlikely to explain these changes.

The overall ICU mortality of 35% is lower than the figures of 48%, 57% and 58% reported in previous UK studies [3-6], and is more similar to reports from New Zealand and other European countries, where seven studies have reported mortalities of 40% or less [10-16]. This apparent improvement in outcome may be due to improved clinical practice (supported perhaps by the fall in admissions only after CPR, which were 7, 13 and 25% in earlier studies [3,5,6]), but may also be due to differing admission policies and also the potential for bias in the previous UK studies. These studies were all small (maximum 62 cases) and two were based in single centres and may, therefore, not be representative of the UK as a whole. The much higher ultimate hospital mortality emphasises the value of this as a more relevant outcome measure. Nevertheless, that approximately 50% of patients survived to leave hospital vindicates the use of ICU resources in the management of patients with CAP. The much higher ultimate hospital mortality is not unique to CAP [7].

The small rise in mortality between the two periods of the study may be because sicker patients were admitted or because of the increase in the proportion of elderly cases in whom comorbidity may be more common and the decision not to augment treatment might be taken at a different threshold to that in younger, fitter individuals. Hospital care, especially identification of the severely ill patient, prior to ICU admission may affect outcome [17,18]. It is not possible to comprehensively assess pre-ICU care from this database. The fall in the prior CPR frequency and the low CPR frequency compared to previous UK studies [3,5,6], might suggest better pre-ICU management. The increase in the pre-ICU length of stay might, however, suggest delay in ICU referral. The admission of more than half of cases within 48 hours of hospital admission supports previous research suggesting that patients with severe illness are usually severely ill at hospital admission and it is at this time that alertness to severity of illness markers is especially important. In previous studies, the proportion of patients admitted early to the ICU has varied markedly. In a Spanish study [19], 80% reached the ICU within 24 hours of hospital admission, whereas in a British multicentre study [6], only 48% reached the ICU within this time. The latter figure is similar to the present case series and may reflect a difference in practice between countries, but may also be artefactually related to the small number in such previous studies. The higher mortality in those admitted later to the ICU is a cause for concern. The precise reasons for this cannot be identified from this database, but late referral could include failure to recognise markers of severe illness [17,18], high use of intermediate care facilities, high ICU bed occupancy limiting ICU access and shortage of ICU beds in the UK. Some late referrals may have had nosocomial pneumonia, which is discussed in detail later. A detailed analysis of factors, including existing severity scoring models, related to outcome is the subject of a separate paper in preparation.

The main weaknesses of this study are its retrospective design and, in particular, that patients with CAP were not separately identified. While it is not possible to be certain that some patients with other diagnoses (such as, nosocomial pneumonia, pneumonia in the immuno-compromised and non-pneumonic exacerbations of chronic obstructive pulmonary disease (COPD)) might not have been included, we believe that the steps taken to exclude such cases and the large size of the study population mean that any such cases that were mistakenly still included are unlikely to have significantly biased the study findings. In particular, the exclusion of postoperative cases and those with specified diagnoses linked with immunosuppression is likely to have minimised inclusion of nosocomial cases and pneumonia in the immuno-compromised; however, nosocomial pneumonia arising in patients admitted for another medical reason might still have been included, but such numbers are likely to be small. If such cases have been inadvertently included, this is most likely to have been in those only admitted to the ICU after seven days in hospital. That we have been successful in excluding even this group is suggested by the absence of any rise in the frequency of cases with secondary admission reasons of myocardial infarction, cardiac failure and COPD in those admitted to the ICU only after two days compared to the less than two days group. The high frequency of a raised PaCO_2 _is not in our opinion a sign of misclassification of patients with exacerbations of COPD. In a recent study, 21% of CAP patients had a PaCO_2 _of >45 mmHg at hospital admission [20], a proportion that is likely to be significantly higher in those admitted to the ICU. The low frequency of 'obstructive airways disease' as a secondary reason for ICU admission also suggests that large numbers of non-pneumonic COPD exacerbations have not been included.

## Conclusion

CAP makes up a small, but significant and rising, proportion of adult ICU admissions. Survival of over half of all cases vindicates the use of ICU facilities in CAP management. Nevertheless, overall mortality remains high, especially in those admitted later in their hospital stay. More widespread use of supports to clinical severity assessment, whether disease specific – for example, Confusion, Urea nitrogen, Respiratory rate, Blood pressure, 65 years of age and older (CURB-65) [21] – or generic – for example, Early Warning Score (EWS) [22] – is to be encouraged.

## Key messages

• 	Community acquired pneumonia accounts for 6% of admissions to adult, general ICUs in the UK.

• 	Mortality was high, with 35% of admissions not surviving the ICU stay and 49% not surviving to leave hospital.

• 	Mortality increased with increasing time in hospital prior to ICU admission.

## Abbreviations

APACHE = Acute Physiology and Chronic Health Evaluation; APS = Acute Physiology Score; CAP = community-acquired pneumonia; CMP = Case Mix Programme; COPD = chronic obstructive pulmonary disease; CPR = cardiopulmonary resuscitation; HDU = high dependency unit; ICNARC = Intensive Care National Audit & Research Centre; ICU = intensive care unit.

## Competing interests

The authors declare that they have no competing interests.

## Authors' contributions

CW performed the analyses. MW, CW and DH drafted the manuscript. All authors contributed to the design and interpretation of the study and critical revision of the manuscript, and have read and approved the final manuscript.

## Supplementary Material

Additional File 1 Additional file 1 is a table providing a comparison of demographics and physiology for admissions with community acquired pneumonia by length of stay in hospital before admission to ICU. Click here for file
