# Supplementary material for: Community-acquired pneumonia on the intensive care unit: secondary analysis of 17,869 cases in the ICNARC Case Mix Programme Database
Source: Crit Care. 2006 Jun 16;10(Suppl 2):S1. doi: 10.1186/cc4927 (PMC3226135; doi:10.1186/cc4927)
Supplement: Additional File 1 — is a table providing a comparison of demographics and physiology for admissions with community acquired pneumonia by length of stay in hospital before admission to ICU. [file cc4927-S1.doc]

# Additional data file 1: Comparison of demographics and physiology for admissions with community acquired pneumonia by length of stay in hospital before admission to ICU

|  | **<2 days** | | **2 – 7 days** | | **>7days** | | **p-value** |
| --- | --- | --- | --- | --- | --- | --- | --- |
|  | **N** | **%** | **N** | **%** | **N** | **%** |  |
| Age, years  < 16  16 – 24  25 – 34  35 – 39  40 – 44  45 – 54  55 – 64  65 – 74  75+ | 228  310  523  809  1,315  2,037  2,770  2,064  226 | 2.2  3.0  5.1  7.9  12.8  19.8  26.9  20.1  2.2 | 69  89  158  290  455  706  1,087  806  93 | 1.8  2.4  4.2  7.7  12.1  18.8  29.0  21.5  2.5 | 23  69  128  168  358  620  1,061  876  99 | 0.7  2.0  3.8  4.9  10.5  18.2  31.2  25.7  2.9 | <0.001 |
| Sex  Female  Male | 4,482  5,800 | 43.6  56.4 | 1,586  2,167 | 42.3  57.7 | 1,327  2,075 | 39.0  61.0 | <0.001 |
| Source of admission  Ward, same hospital  A&E, same hospital, clinic or home  Other intermediate care area, same hospital  HDU, same hospital  ICU, same hospital  ICU, other hospital  HDU, other hospital  Other hospital (not ICU or HDU) | 4,088  2,759  427  527  37  1,213  143  1,078 | 39.8  26.9  4.2  5.1  0.4  11.8  1.4  10.5 | 2,897  45  119  606  47  22  5  12 | 77.2  1.2  3.2  16.1  1.3  0.6  0.1  0.3 | 2,451  28  86  662  109  46  3  17 | 72.0  0.8  2.5  19.5  3.2  1.4  0.1  0.5 | <0.001 |
| CPR within 24 hours prior to admission  Yes  No | 675  9,588 | 6.6  93.4 | 209  3,536 | 5.6  94.4 | 168  3,228 | 4.9  95.1 | <0.001 |
| Past medical history of  Severe liver problems  Very severe cardiovascular disease  Steroid treatment  Severe respiratory problems  Chronic renal replacement therapy | 98  175  325  841  90 | 1.0  1.7  3.2  8.2  0.9 | 60  82  103  237  52 | 1.6  2.2  2.7  6.3  1.4 | 58  70  92  162  84 | 1.7  2.1  2.7  4.8  2.5 | <0.001  0.134  0.403  <0.001  <0.001 |
| Secondary reason for admission:  Pre-existing:  Obstructive airways disease  Trauma  Diabetes mellitus  Chronic neuromuscular disorders  Alcoholic cirrhosis  Chronic renal failure  Pulmonary fibrosis or fibrosing alveoli  Asthma attack  Inhalation pneumonitis (gastrointestinal contents)  Occurring as CAP complication:  Septic shock/septicaemia  Pleural effusion  Could be pre-existing or complication:  Acute renal failure  Cardiac dysrhythma  Cardiac failure  Cardiogenic shock  Acute myocardial infarction  Pulmonary embolus (thrombus)  Status epilepticus or uncontrolled seizures  Non-cardiogenic pulmonary oedema (ARDS)  Other/none | 943  53  83  62  68  90  61  140  24  730  38  503  126  398  33  129  24  63  73  6,641 | 9.2  0.5  0.8  0.6  0.7  0.9  0.6  1.4  0.2  7.1  0.4  4.9  1.2  3.9  0.3  1.3  0.2  0.6  0.7  64.6 | 214  47  16  17  26  38  27  27  11  240  31  183  65  119  15  46  16  19  38  2,558 | 5.7  1.3  0.4  0.5  0.7  1.0  0.7  0.7  0.3  6.4  0.8  4.9  1.7  3.2  0.4  1.2  0.4  0.5  1.0  68.2 | 120  18  15  16  29  51  23  8  19  228  52  169  52  110  10  33  15  8  39  2,387 | 3.5  0.5  0.4  0.5  0.9  1.5  0.7  0.2  0.6  6.7  1.5  5.0  1.5  3.2  0.3  1.0  0.4  0.2  1.1  70.2 | <0.001 |
| Systolic blood pressure at admission, mmHg  < 60  60 – 69  70 – 79  80 – 89  90 – 99  100 – 109  110 – 119  120 – 179  ≥ 180 | 88  176  333  544  851  2,475  1,136  4,309  370 | 0.9  1.7  3.2  5.3  8.3  24.1  11.0  41.9  3.6 | 23  47  85  159  251  896  415  1,700  177 | 0.6  1.3  2.3  4.2  6.7  23.9  11.1  45.3  4.7 | 21  36  72  169  260  809  369  1,521  145 | 0.6  1.1  2.1  5.0  7.6  23.8  10.8  44.7  4.3 | <0.001 |
| Heart rate at admission, beats min^‑1^  < 50  50 – 99  100 – 109  110 – 119  120 – 139  140 – 154  ≥ 155 | 41  4,589  1,384  1,341  1,914  660  353 | 0.4  44.6  13.5  13.0  18.6  6.4  3.4 | 10  1,579  553  540  734  213  124 | 0.3  42.1  14.7  14.4  19.6  5.7  3.3 | 21  1,512  485  480  612  182  110 | 0.6  44.4  14.3  14.1  18.0  5.3  3.2 | 0.004 |
| Highest respiratory rate if not mechanically ventilated on admission (breaths min^-1^)  < 15  15 – 19  20 – 24  25 – 29  30 – 34  ≥ 35  Mechanically Ventilated | 889  83  279  501  712  1,636  6,182 | 8.6  0.8  2.7  4.9  6.9  15.9  60.1 | 401  42  182  282  364  799  1,683 | 10.7  1.1  4.8  7.5  9.7  21.3  44.8 | 326  39  120  263  345  711  1,598 | 9.6  1.1  3.5  7.7  10.1  20.9  47.0 | <0.001 |
| Lowest total Glasgow Coma Score if not sedated/paralysed  15  11 – 14  7– 10  4 – 6  3  Sedated/Paralysed | 3,565  804  413  204  557  4,739 | 34.7  7.8  4.0  2.0  5.4  46.1 | 1,451  365  162  71  186  1,518 | 38.7  9.7  4.3  1.9  5.0  40.1 | 1,318  387  189  91  159  1,258 | 38.7  11.4  5.6  2.7  4.7  37.0 | <0.001 |
| Lowest PaO_2_/FIO_2_, kPa  Non-ventilated  < 1.0  1.0 – 2.9  3.0 – 3.9  4.0 – 4.9  5.0 – 6.9  ≥ 7.0  Ventilated  < 1.0  1.0 – 2.9  3.0 – 3.9  4.0 – 4.9  5.0 – 6.9  ≥ 7.0 | 110  1,277  331  171  454  22  787  4,779  1,280  534  479  58 | 1.1  12.4  3.2  1.7  4.4  0.2  7.7  46.5  12.4  5.2  4.7  0.6 | 68  640  142  65  175  13  300  1,724  349  160  104  13 | 1.8  17.1  3.8  1.7  4.7  0.3  8.0  45.9  9.3  4.3  2.8  0.3 | 35  574  155  87  209  10  205  1,476  314  181  142  14 | 1.0  16.9  4.6  2.6  6.1  0.3  6.0  43.4  9.2  5.3  4.2  0.4 | <0.001  <0.001 |
| Lowest pH  Non-ventilated  < 7.15  7.15 – 7.24  7.25 – 7.32  7.33 – 7.4  ≥ 7.5  Ventilated  < 7.15  7.15 – 7.24  7.25 – 7.32  7.33 – 7.4  ≥ 7.5 | 122  239  464  1,517  23  1,830  1,762  1,651  2,601  73 | 1.2  2.3  4.5  14.8  0.2  17.8  17.1  16.1  25.3  0.7 | 38  85  193  776  11  497  624  637  876  16 | 1.0  2.3  5.1  20.7  0.3  13.2  16.6  17.0  23.3  0.4 | 27  73  134  812  24  320  427  557  994  34 | 0.8  2.1  3.9  23.9  0.7  9.4  12.6  16.4  29.2  1.0 | <0.001  <0.001 |
| Associated PaCO_2_, kPa  Non-ventilated  < 3.0  3.0 – 3.9  4.0 – 4.4  4.5 – 5.4  5.5 – 5.9  6.0 – 6.4  ≥ 6.5  Ventilated  < 3.0  3.0 – 3.9  4.0 – 4.4  4.5 – 5.4  5.5 – 5.9  6.0 – 6.4  ≥ 6.5 | 21  173  207  1,032  197  133  602  41  159  256  1,720  626  673  4,442 | 0.2  1.7  2.0  10.0  1.9  1.3  5.9  0.4  1.5  2.5  16.7  6.1  6.5  43.2 | 10  64  99  488  110  86  246  14  66  106  558  230  249  1,427 | 0.3  1.7  2.6  13.0  2.9  2.3  6.6  0.4  1.8  2.8  14.9  6.1  6.6  38.0 | 10  59  84  459  107  91  260  8  52  85  545  208  227  1,207 | 0.3  1.7  2.5  13.5  3.1  2.7  7.6  0.2  1.5  2.5  16.0  6.1  6.7  35.5 | 0.045  0.013 |
| Base excess, mmol  Non-ventilated  < -16  -16 – -13  -12 – -9  -8 – -5  ≥ -4  Ventilated  < -16  -16 – -13  -12 – -9  -8 – -5  ≥ -4 | 34  57  156  393  1,725  410  547  942  1,423  4,595 | 0.3  0.6  1.5  3.8  16.8  4.0  5.3  9.2  13.8  44.7 | 16  24  64  137  862  110  136  315  545  1,544 | 0.4  0.6  1.7  3.7  23.0  2.9  3.6  8.4  14.5  41.1 | 13  7  47  110  893  84  75  188  338  1,647 | 0.4  0.2  1.4  3.2  26.2  2.5  2.2  5.5  9.9  48.4 | <0.001  <0.001 |
| Serum sodium, mmol l^-1^  < 120  120 – 129  130 – 134  135 – 144  145 – 149  150 – 154  155 – 159  ≥ 160 | 168  1,165  2,353  3,282  2,912  264  89  49 | 1.6  11.3  22.9  31.9  28.3  2.6  0.9  0.5 | 34  419  799  1,102  1,155  162  52  30 | 0.9  11.2  21.3  29.4  30.8  4.3  1.4  0.8 | 36  327  768  871  1,097  198  69  36 | 1.1  9.6  22.6  25.6  32.2  5.8  2.0  1.1 | <0.001 |
| Serum potassium, mmol l^-1^  < 2.5  2.5 – 2.9  3.0 – 3.4  3.5 – 4.9  5.0 – 5.9  6.0 – 6.9  ≥ 7 | 120  594  2,107  5,625  1,365  381  90 | 1.2  5.8  20.5  54.7  13.3  3.7  0.9 | 62  258  808  2,061  426  116  22 | 1.7  6.9  21.5  54.9  11.4  3.1  0.6 | 52  220  704  1,862  453  93  18 | 1.5  6.5  20.7  54.7  13.3  2.7  0.5 | <0.001 |
| Serum urea, mmol l^-1^  < 4.0  4.0 – 7.9  8.0 – 14.9  15.0 – 29.0  ≥ 30.0 | 722  3,529  3,008  2,394  629 | 7.0  34.3  29.3  23.3  6.1 | 384  1,384  968  784  233 | 10.2  36.9  25.8  20.9  6.2 | 283  1,246  998  633  242 | 8.3  36.6  29.3  18.6  7.1 | <0.001 |
| Serum creatinine, μmol dl^-1^  < 60  60 – 109  110 – 169  ≥ 170 | 937  3,938  2,356  3,051 | 9.1  38.3  22.9  29.7 | 454  1,654  772  873 | 12.1  44.1  20.6  23.3 | 526  1,468  639  769 | 15.5  43.2  18.8  22.6 | <0.001 |

A&E: accident & emergency; ARDS: acute respiratory distress syndrome; CAP: community-acquired pneumonia; CPR: cardiopulmonary resuscitation; HDU: high dependency unit; ICU: intensive care unit

Note: admissions were excluded from additional data file 1 if they were a readmission of the same patient within the same hospital stay or if they were missing data to calculate the length of stay in hospital before admission to ICU
